# New sesquiterpene coumarin from the roots of *Ferula latisecta*

**Published:** 2012

**Authors:** Mehrdad Iranshahi, Farjad Amanolahi, Bernd Schneider

**Affiliations:** 1 *Biotechnology Ressearch Center, School of Pharmacy, Mashhad University of Medical Sciences, Mashhad, I. R. Iran*; 2 *Max Planck Institute for Chemical Ecology, Beutenberg Campus, Jena, Germany*

## Abstract

**Objective:** The genus of *Ferula* belongs to the tribe Peucedaneae, subfamily of Apioideae and family of Umbelliferae with 133 species distributed throughout the Mediterranean area and central Asia, especially in the former USSR and neighboring countries such as Iran. The popular Persian name of the most of these species is “Koma”. In this research we tried to isolate and elucidate the structure of new sesquiterpene in the root of *Ferula latisecta (F. latisecta).*

**Materials and Methods:** Dried and powdered roots of *F. latisecta* were extracted with CH_2_Cl_2_ using a Soxhlet apparatus. The extract was concentrated *in vacuo *to give a red extract. The extract was subjected to column chromatography on silica gel. ^1^H NMR, ^13^C NMR, DEPT, ^1^H-^1^H COSY, HMBC, HSQC, and NOESY spectra were the methods we used to elucidate the structure of new sesquiterpene in this plant.

**Results:** One new sesquieterpene coumarin, namely Latisectin and IUPAC name [1-(2-Hydroxy-4-methoxy-phenyl)-3,4,8,12-tetramethyl-trideca-4,7,11-trien-1-one ] , together with one known compound , Kopetdaghin C, were isolated from the root of *F. latisecta.*

**Conclusion:** In this research the structure of one new and one known sesquiterpene in the root of *F. latisecta* was elucidated.

## Introduction

The genus of *Ferula *belongs to the tribe Peucedaneae, subfamily of Apioideae and family of Umbelliferae with 133 species distributed throughout the Mediterranean area and central Asia, especially in the former USSR and neighboring countries such as Iran (Evans, 1989 ▶; Mozaffarian, 1983 ▶; Heywood, 1985 ▶). More than 70 species of *Ferula *have already been investigated phytochemically (Diab et al., 2001 ▶; Iranshahi et al., 2004a ▶; Abd El-Razek et al., 2003 ▶). Several species of this genus have been used in folk medicine (Chen et al., 2000 ▶). The Iranian flora comprises 30 species of *Ferula* of which 15 are endemic. (Mozaffarian, 1983 ▶, 1996). The popular Persian name for the most of these species is “Koma” (Mozaffarian, 1996 ▶).

The chemistry of this genus has been studied by many investigators and is well documented as a good source of biologically active compounds such as sesquiterpene derivatives (Ahmed et al., 2001 ▶; Ahmed, 1999 ▶; Valle et al., 1987; Iranshahi et al., 2004b ▶, 2007, 2008; Shahverdi et al., 2006). daucanes, humulanes, himachalanes, germacranes, eudesmanes, and guainanes (Gonzalez and Barrera, 1995 ▶; Appendino et al., 1997 ▶; Kojima et al., 1999 ▶, 2000; ▶ Chen et al., 2000 ▶; Su et al., 2000a ▶,b ▶) Sesquiterpene derivatives, especially sesquiterpene coumarins, were stored in the roots of the plants; therefore the roots are a better source for isolating sesquiterpene coumarins than the aerial parts.

*F. latisecta* Rech. f. & Aellen. Is a plant endemic to Iran (Hedge et al., 1982 ▶) and no phytochemical studies of this species have been reported to date. One report showed an inhibitory effect of *F. latisecta* root extract on gram positive bacteria and candida albicans (Iranshahi et al., 2008 ▶). One of traditional usage of this plant is related to its anti-parasitic effects. Also because of sulfide compounds in this plant, it has been used to treat gastro-intestinal disorders in domestic animals. The aim of this study is isolation and elucidation the compounds in the root of this plant. 

## Materials and Methods

**Plant material**

The roots of *F. latisecta* were collected from the Hezarmasjed Mountains, Khorasan Razavi province, Iran, in April 2011. The plant material was identified by Mohammad Reza Joharchi, Ferdowsi University of Mashhad Herbarium (FUMH). A voucher specimen (No. 1004) has been deposited at the herbarium of School of Pharmacy, Mashhad University of Medical Sciences.

**General experimental procedures**

NMR spectra were measured using a Bruker DRX 500 (Bruker Biospin, Rheinstetten, Germany). ^1^H NMR, ^13^C NMR, DEPT, ^1^H-^1^H COSY, HMBC, HSQC, and NOESY spectra were measured using an inverse-detection probe (5 mm). The operating frequencies were 500.13 MHz for acquiring ^1^H NMR and 125.75 MHz for ^13^C NMR spectra. Samples were measured at 300 K in CDCl_3_ with TMS as the internal standard. Column chromatography was conducted with silica gel 230-400 mesh (Merck, Berlin, Germany). Preparative Thin Layer Chromatography (TLC) was performed on GF254s plates (20×20 cm, Merck, Berlin , Germany) and observation of the plates was carried out under UV CAMAG spectrometer (254 nm) (Evans, 1989 ▶ ; Iranshahi et al., 2004b ▶).

**Extraction and isolation**

Dried and powdered roots of *F. latisecta *(500 g) were extracted with CH_2_Cl_2_ using a Soxhlet apparatus. The extract was concentrated *in vacuo *to give a red extract (18 g) and then they subjected to column chromatography on silica gel (5×50 cm) using petroleum ether with increasing volumes of EtOAc. petrol (1 L) , petrol:EtOAc (98:2, 1.5 L) , (96:4, 1.5 L) , (94:6, 1.5 L) , (92:8, 1.5 L) , (90:10, 3 L) , (88:12, 1.5 L) , (86:14, 1.5 L) , (84:16, 1.5 L) , (82:18, 1.5 L) , (80:20, 3 L) , (75:25, 2 L) , (70:30, 3 L) , (65:35, 2 L) , (60:40, 3 L) , (50:50, 2 L) , (40:60, 2 L) , (30:70, 2 L) , (20:80, 2 L) , (10:90, 2 L) and EtOAc (3 L)). The fractions were compared by TLC, and those giving similar spots were combined and thirteen fractions were finally obtained. Fractions required more purification with PTLC (silica gel using petrol: EtOAc, in different ratio, 20×20 cm, glass plates , each plate was run two times). After further purification, each purified fraction was kept in glass vial without any solvent and reserved in freezer in-18 ^º^C, but some of them were unstable and finally only 2 fractions were obtained for structure identification.

## Results

**Figure 1.**
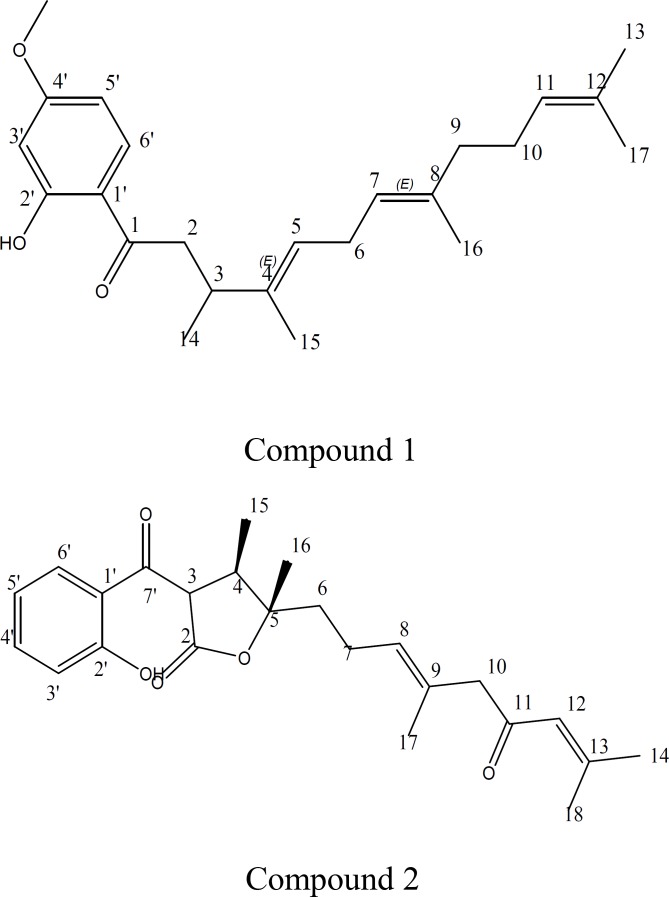
Chemical structure of two compounds isolated from *Ferula latisecta*

Normal-phase column chromatography of the dichloromethane extract of roots, followed by preparative TLC, afforded two natural products (Figure 1).

Compound 1, Latisectin, is a new compound from *F.latisecta*. The molecular formula of this compound C_24_H_34_O_3_, was established by HREIMS (m/z 370.251289 , calc 370.25095). Its structure was established with ^1^H and ^13^C NMR spectra (Table 1). Compound 2, Kopetdaghin C, has been known before in *Dorema kopetdaghin *(Iranshahi et al., 2007b ▶)*,* and final structure of Kopetdaghin C is described in details in Table 2.

**Table 1:** ^1^H NMR (500 MHz) and ^13^C NMR (125 MHz) data for compound 1 (CDCl_3_)^a ^.

	** Compound 1**	
**Position**	**δ**_H_	**δ**_c_
**1**	--------	204.5
**2**	2.97 d (5.8)	43.8
**3**	2.77 dt (8.5 ,7)	39.6
**4**	--------	137.8
**5**	5.14 t (7)	123.4
**6**	2.64 dd (7.7) like t	26.7
**7**	5.02 t (7)	122.8
**8**	--------	135.2
**9**	2.96 m	39.6
**10**	2.04 m	26.7
**11**	5.08 t (7)	124.3
**12**	--------	131.3
**13**	1.67 s	25.8
**14**	1.07 d (6)	19.4
**15**	1.63 s	13.4
**16**	1.59 s	16.0
**17**	1.60 s	17.7
**18**	--------	--------
**1'**	--------	113.8
**2'**	--------	165.6
**3'**	6.41 s	100.9
**4'**	--------	165.9
**5'**	6.41 d (8.5)	107.4
**6'**	7.34 d (8.5)	131.8
**7'**	--------	--------
**OH**	12.46 s	--------
**OCH**_3_	3.83 s	55.5

**Table 2:** ^1^H NMR (500 MHz) and ^13^C NMR (125 MHz) data for compound 2 (CDCl_3_)^a^

	** Compound 2 ** b		** Compound 2 ** c	
**Position**	**δ**_H_	**δ** c	**δ**_H_	**δ** c
**1**	--------	--------	--------	--------
**2**	--------	171.4	--------	171.1
**3**	4.27 d (11.8)	54.9	4.22 d (12)	54.5
**4**	3.18 dq (11.8)	41.6	3.15 dq (12.7)	41.2
**5**	--------	88.4	--------	87.6
**6**	1.83 s	39.8	1.80 s	39.3
**7**	2.32 s	22.9	2.32 s	22.5
**8**	5.28 t (6.6)	128.1	5.25 t (6)	127.6
**9**	--------	131.3	--------	130.8
**10**	3.10 s	55.6	3.05 s	55.1
**11**	--------	199.5	--------	199.1
**12**	6.15 s	123.3	6.10 brs	122.9
**13**	--------	156.4	--------	155.9
**14**	1.92 s	28.1	1.88 s	27.7
**15**	1.1d (6.6)	13.8	1.06 d (7)	13.4
**16**	1.39 s	20.9	1.35 s	20.5
**17**	1.70 s	16.9	1.65 s	16.5
**18**	2.19 s	21.1	2.15 s	20.7
**1'**	--------	114.3	--------	113.9
**2'**	--------	167.4	--------	166.9
**3'**	6.48 d (2.1)	101.3	6.44 d (2)	100.9
**4'**	--------	166.7	--------	166.2
**5'**	6.45 dd (9.5)	108.8	6.50 dd (9.2)	108.3
**6'**	7.71 d (9)	133.9	7.67 d (9)	132.9
**7'**	--------	196.3	--------	195.9
**OH**	12.52 s	--------	12.46 s	--------
**OCH**_3_	3.90 s	56.1	3.86 s	55.7

a J values are in parentheses and reported in Hz; assignments were confirmed by ^1^H-^1^H COSY, HMQC, HMBC and NOESY experiments.

b These data are related to previous study by Iranshahi et al., 2007b.

c These data are related to our study.

## Discussion

The ^13^C NMR of compound 1 resonance had 24 carbon signals that are similar to sesquiterpene structure. HSQC spectrum classified the carbon signals to four aliphatic methylenes at δ_c _37.3 (C-8’), 30.6 (C-1’), 25.4 (C-7’), 37.6 (C-2’), 65.3 (C-11’; characteristic for an oxygenated methylene) , and 113.4 (C-15’), and six methines at 137.8 (C-4), 135.2 (C-8), 131.3 (C-12), 113.8 (C-1'), 165.6 (C-2') and 165.9 (C-4') and five methyls at 25.8 (C-13), 19.4 (C-14), 13.4 (C-15), 16 (C-16) and 17.7 (C-17). Moreover, we can see signal of one ketone group at 204.5 and four tertiary carbon at 43.8 (C-2), 26.7 (C-6), 39.6 (C-9) and 26.7 (C10) in ^13^C NMR. The 1H NMR spectrum of 1 showed resonances characteristic for five methyl singlets at δ_H_ 1.67 (H-13), 1.07 (H-14), 1.63 (H-115), 1.59 (H-16), 1.60 (H-17) and one methoxy group at 3.83. Three aromatic protons at δ_H_ 6.41 (H-3'), 6.41 (H-5') and 7.34 (H-6') suggested the presence of a benzene ring, which was supported by the ^13^C NMR spectrum. 

In the HMBC spectrum, the correlations of H-7 (δ_H_ 5.02) with C-6 (26.7) and C-8 (135.2), H-13 (δ_H_ 1.67) with C-12 (δ_H_ 131.3), H-6’ (δ_H _7.34) with C-1' (δ_c_ 113.8), H-12’ and C-5' (17.4), OCH_3_ (δ_c_ 3.83) with C-4’ (165.9) and OH (12.46) with C-2' ( 165.6) and C-1' ( 113.8) and C-3' ( 100.9) confirmed the structure of compound 1. The proposed structure was further supported by ^1^H-^1^H COSY data. The position of double bond in compound 1 was established from the HMBC spectrum and we saw their signal in ^1^H NMR at 5.14 (H-5), 5.02 (H-7) and 5.08 (H-11).

Therefore according to these findings, we named compound 1 with sesquieterpene structure in IUPAC: 1-(2-Hydroxy-4-methoxy-phenyl)-3,4,8,12-tetramethyl-trideca-4,7,11-trien-1-one.

## References

[B1] Abd El-Razek MH, Ohta S, Hirata T (2003). Terpenoid coumarins of the genus Ferula. Heterocycles.

[B2] Ahmed AA (1999). Sesquiterpene coumarins and sesquiterpenes from Ferula sinaica. Phytochemistry.

[B3] Ahmed AA, Abd El-Razek MH, Nassar MI, Izuma S, Ohta S, Hirata T (2001). Sesquiterpene coumarins from the roots of Ferula assafoetida. Phytochemistry.

[B4] Chen B, Teranishi R, Kawazoe K, Takaishi Y, Honda G, Itoh M, Takeda Y, Kodzhimatov OK (2000). Sesquiterpenoids from Ferula kuhistanica. Phytochemistry.

[B5] Diab Y, Dolmazon R, Bessiere JM (2001). Daucane aryl esters composition from the Lebanese Ferula hermonis Boiss (zallooh root). Flav Fragr J.

[B6] Evans WC (1989). Trease and Evans’ Pharmacognosy.

[B7] Hedge IC, Lamond JM, Rechinger KH (1982). Ferula, in: Flora Iranica. Umbelliferae.

[B8] Heywood VH (1985). Flowering Plants of the World, London, Croom Helm.

[B9] Iranshahi M, Amin GR, Shafiee A (2004a). A new coumarin from Ferula persica. Pharm Biol.

[B10] Iranshahi M, Arfa P, Ramezani M, Jaafari MR, Sadeghian H, Bassarello C, Piacente S, Pizza C (2007a). Sesquiterpene coumarins from Ferula szowitsiana and invitro antileishmanial activity of 7 prenyloxycoumarins against promastigotes. Phytochemistry.

[B11] Iranshahi M, Kalategi F, Rezaee R, Shahverdi AR, Ito C, Furukawa H, Tokuda H, Itoigawa M (2008). Cancer chemopreventive activity of terpenoid coumarins from Ferula species. Planta Med.

[B12] Iranshahi M, Shahverdi AR, Mirjani R, Amin GR, Shafiee A (2004b). Umbelliprenin from Ferula persica roots inhibits the red pigment production in Serratia marcescens. Z. Naturforschung.

[B13] Iranshahi M, Shaki F, Mashlab A, Wessjohan LA (2007b). Kopetdaghins A-E, sesquiterpene derivatives from the aerial parts and the roots of Dorema kopetdaghense. J Nat Prod.

[B14] Mozaffarian V (1983). The Family of Umbelliferae in Iran: Keys and Distribution.

[B15] Mozaffarian V (1996). A Dictionary of Iranian Plant Names.

[B16] Gonzalez AG, Barrera JB (1995). Chemistry and the sources of mono-and bicyclic sesquiterpenes from Ferula species. Progress in the Chemistry of Organic Natural Products.

[B17] Appendino G, Jakupovic J, Alloatti S, Ballero M (1997). Daucane esters from Ferula arrigonii. Phytochemistry.

[B18] Kojima K, Isaka K, Ondognii P, Zevgeegiino O, Davgiin K, Mizukami H, Ogihara Y (1999). Sesquiterpenoid derivatives from Ferula ferulioids III. Chemical and Pharmaceutical Bulletin.

[B19] Kojima K, Isaka K, Ondognii P, Zevgeegiino O, Gombosurengyin P, Davgiin K, Mizukami H, Ogihara Y (2000). Sesquiterpenoid derivatives from Ferula ferulioids. Chemical and Pharmaceutical Bulletin.

[B20] Su BN, Takaishi Y, Honda G, Itoh M, Takeda Y, Kodzhimatov OK, Ashurmetov O (2000). Sesquiterpene coumarins and related derivatives from Ferula pallid. J Nat Prod.

[B21] Su BN, Takaishi Y, Honda G, Itoh M, Takeda Y, Kodzhimatov OK, Ashurmetov O (2000). Sesquiterpene phenylpropanoid and sesquiterpene chromone derivatives from Ferula pallida. J Nat Prod.

